# Patient safety attitude among intensive care unit physicians and nurses: a multi-center study in Egypt

**DOI:** 10.1186/s42506-025-00190-2

**Published:** 2025-05-14

**Authors:** Taghareed A. Elhoseny, Mohamed A. Kandil, Rasha A. Mosallam

**Affiliations:** 1 Department of Health Administration and Behavioural Sciences, High Institute of Public Health, University of Alexandria, Alexandria, Egypt; 2 Department of Emergency Medical Care, Imam Abdulrahman Bin Faisal University, Dammam, Saudi Arabia

**Keywords:** Safety culture, Patient safety, Safety Attitudes Questionnaire, Intensive care unit staff, ICU physicians and nurses, Egypt

## Abstract

**Background:**

Continuous safety culture assessment, especially in high-risk areas as the intensive care unit (ICU) is one of the requirements for patient safety. This study aimed to assess patient safety attitude in the intensive care units of four public hospitals in Egypt, compare it with benchmarking data, and identify opportunities for improvement.

**Methods:**

A cross-sectional design was used. The Safety Attitudes Questionnaire (SAQ) was distributed in March 2024 to a total of 543 physicians and nurses involved in direct patient care in eight ICU of four public hospitals. A response rate of 85% was achieved (65.12% for physicians and 89.28% for nurses). Safety culture score (ranges from 0 to 100) and percent-positive scores (percentage of respondents with a mean score of > 75 where 100 is best) were calculated according to the tool scoring key. The scores were also compared with the benchmarking scores.

**Results:**

The overall mean scale score was 63.7 ± 13.4 and the percentage of positive responses was 58.1%. Job satisfaction was the dimension with the highest total mean and percentage of positive responses (70.0 and 65.1%, respectively). On the other hand, stress recognition was the dimension with the lowest mean score and percentage of positive responses (59.4 ± 23.8 and 53.6%, respectively). Physicians attained a significantly higher total mean scale score for the teamwork climate, job satisfaction, perceptions of management and work conditions dimensions compared to nurses (73.8 ± 10.4, 81.7 ± 14.6, 72.5 ± 19.4, 70.6 ± 18.1 for physicians compared to 61.0 ± 15.4, 68.4 ± 23.0, 60.8 ± 22.4, 61.8 ± 22.3 for nurses, respectively) (*p* < 0.05) while “stress recognition’’ was significantly higher among nurses (mean scale score for nurses was 60.4 ± 23.6, *p* < 0.05 compared to 52.0 ± 24.0, *p* < 0.05 for physicians). The dimensions “teamwork climate’’, “safety climate’’ and “stress recognition’’ attained lower scores in the current study compared to the benchmark data.

**Conclusion:**

The dimensions “teamwork climate’’, “safety climate’’, and “stress recognition’’ attained relatively low scores which calls for interventions such as team trainings, limitation of work hours and senior executive safety rounds. Physicians had significantly higher scores than nurses in four out of six domains which needs further research to identify the reasons and plan the appropriate improvement strategies. Future studies should track changes over time.

## Introduction

It is estimated that worldwide over 10% of inpatients experience adverse events half of which are preventable. Medical errors result in approximately 3 million deaths and 64 million Disability-Adjusted Life Years (DALYs) annually [1–3]. Adverse events impose a significant burden on the healthcare finance due to the increased resource use and lost productivity and lives. In low-to-middle income countries the situation is alarming as it was estimated that a quarter of hospitalizations result in harm and that one in every twenty two people die due to unsafe practices [4]. Patient safety has become a worldwide priority. A strong safety culture is a fundamental element of a successful patient safety system. The Joint Commission defines safety culture as the “product of individual and group beliefs, values, attitudes, perceptions, competencies, and patterns of behavior that determine the organization’s commitment to quality and patient safety.” [5]. These values and beliefs are intended to shape workers’ behavior helping them minimize or prevent harm to patients during care [6, 7].

Safety culture is multidimensional. A systematic review of 694 studies that assessed safety culture in hospitals defined 11 themes: leadership; perceptions of safety; teamwork and collaboration; safety systems; prioritization of safety; resources and constraints; reporting and just culture; openness; learning and improvement; awareness of human limits; and wellbeing [8]. Although both terms “safety culture” and “safety climate” are often used interchangeably, safety climate is a narrower term focusing on the staff’s current perceptions about supervision, resources, and policies of their organization [9]. There are multiple tools for the measurement of safety culture. A review of the available tools for the assessment of safety culture showed that the SAQ is the most popular and evaluated tool for measuring health care safety culture. Furthermore, it can be used to assess the attitudes of different types of staff as physicians and nurses [10].

The Joint Commission urges regular assessment of safety culture to uncover unsafe conditions that may jeopardize the quality of patient care [11]. In Egypt, The General Authority for Healthcare Accreditation (GAHAR) requires healthcare leaders and managers to create a culture of safety and quality within the facility to be accredited. In its intent section, the accreditation standards book refers to the need for a strong safety culture that can encourage voluntary reporting of errors by reducing the fear of blame. This makes spotting deficiencies possible along with the subsequent planning of corrective actions. In order to gather evidence for their assessment, surveyors review the records of leaders’ safety rounds during the leadership interview session to assess safety culture, and interview staff to check their support for safety culture quality initiatives [12]. In Egypt, research on healthcare safety culture was carried out both in hospitals and in primary care units [13–16]. These studies were conducted in teaching and in general hospitals. The critical care units are considered to be high risk areas with specialized medical devices and severe cases in which patient safety and safety culture is of special importance [17]. The SAQ was administered to 8646 ICU professionals in the UK, USA, and New Zealand and the data acts as international benchmarking data that allows safety culture to be compared across healthcare organizations and countries [18]. Despite this, there is a lack of research on assessing the safety culture in critical care units in Egypt. Our study aimed to measure the safety climate in the intensive care units of four public hospitals in Egypt and to compare it with benchmarking data to identify opportunities for improvement.

## Methods

### Study design and setting

This study was conducted at the critical care units of four public hospitals in two governorates in northern Egypt (Alexandria and El-Beheira) in March 2024. Hospitals with intensive care units in both governorates were recruited for the study and only those who provided administrative approval were included. It is a cross-sectional study that was conducted in the eight ICU units. The first hospital is a 100-bed public pediatric hospital affiliated with the Ministry of Health and Population (MOHP). It has a 50-bed Neonatal Intensive Care Unit (NICU) and a 16-bed pediatric intensive care unit which provides services for children from birth till 16 years old. The second hospital is a 300-bed public general hospital affiliated with the MOHP. It has a 24-bed general intensive care unit, a six-bed cardiac care unit, a six-bed poisoning ICU and a 12-bed pediatric intensive care unit. The third hospital is a 200-bed public general hospital affiliated with the MOHP. It has a 14-bed adult intensive care unit and a five-bed intensive cardiac care unit. The fourth hospital is a 350-bed public general university hospital. It has an 18-bed adult intensive care unit and a 30-bed intensive cardiac care unit.

### Sample size and sampling technique

All ICU physicians and nurses working in the ICU units in the four hospitals were invited to participate. Those who have been working for less than 6 months were excluded (one physician and fourteen nurses). A total of 457 nurses and 86 physicians (175 nurses and 40 physicians in the first hospital, 190 nurses and 28 physicians in the second hospital, 43 nurses and 10 physicians in the third hospital, and 49 nurses and 8 physicians in the fourth hospital) were invited. Fifty six out of 86 physicians responded (response rate 65.12%) and 408 out of 457 nurses responded (response rate 89.28%).

### Data collection tools questionnaire

A structured self-administrated paper-based questionnaire was used that is composed of two parts:

**First Part**

Personal and background information: including age, sex, experience, working area, and profession (physician or nurse).

**Second Part**

Attitude of healthcare workers towards safety culture was assessed using the Safety Attitude Questionnaire [18]. The six safety culture domains—teamwork environment, safety climate, job satisfaction, stress recognition, perception of management, and working conditions—are evaluated using the SAQ. Respondents must rate their degree of agreement with each item on a 5-point Likert scale, with 1 denoting strong disagreement and 5 denoting strong agreement. Two items—6 and 13—have negative wording, they hence are reverse graded. The scores for each item were calculated by converting the 5-point Likert scale into a 100-point scale as follows: 1 = 0, 2 = 25, 3 = 50, 4 = 75, and 5 = 100.

To get a score between 0 and 100, the scores of all the items in the same dimension were then added together and divided by the total number of elements in that dimension. The same formula was used to determine the respondent’s overall score for the patient safety culture level. For determining the percentage of positive responses (percent agreement) the total frequency of agree slightly (4) and agree strongly (5) were summed then divided by the total number of respondents, and multiplied by 100. A response was considered positive if the overall percentage was greater than or equal to 75; a negative response was indicated if it was less than 75. Regarding negative responses, negatively worded items (6 and 13) were reverse coded to indicate a positive perception (2 = agree slightly, 1 = agree strongly) [18].

The questionnaire was translated to Arabic by two of the researchers who are native Arabic speakers, fluent in English, and held doctoral degrees in hospital administration. The two translations were compared, minor differences were observed and discussed until a consensus was reached. Back translation to English was tasked to an academic coworker with the same specialty. The back translation was compared to the original questionnaire by the three-member group who found a high degree of conformity except for three questions in which a few words differed but did not alter the meaning. The questionnaire was piloted on 6 nurses to test for its language clarity and comprehensibility and they reported no difficulties. Completion time was about 15 min. Since the tool was translated to Arabic, content validity of the questionnaire was performed. A validation review questionnaire was sent independently to four experts to assess the validity of items on a four-point scale of relevance (1 = not relevant, 2 = somewhat relevant, 3 = quite relevant, and 4 = highly relevant). Item content validity index (I-CVI) was calculated as

$$\text{I-CVI}=\frac{\text{Number of judges who rated the item with }3\text{ or }4}{\text{Total number of judges}}$$

Scale content validity index (S-CVI) was determined as the proportion of items rated as either 3 or 4.. A CVI score of 0.80 or better indicates good content validity. Cronbach’s alpha value was calculated to test for internal consistency. The I-CVI of the items ranged from 0.75 to 1, while the S-CVI was 0.81.

### Statistical analysis

The IBM Statistical Package for Social Sciences (SPSS) software (version 23) was used to code and enter the data [19]. Descriptive data was presented using mean ± SD, frequencies, and percentages. The statistical significance of the categorical data was examined using the chi-square test and Fisher’s exact test. When necessary, sample *t*-tests or analysis of variance (ANOVA) tests were used to compare means and investigate the relationship between patient safety culture and the independent factors. Results were considered statistically significant if the *P*-value fell below 0.05.

## Results

Physicians were mostly females (57.1%), in the age group 30–39 (64.2%), working 3– < 5 years in the specialty (37.5%), 3– < 5 years in the unit (62.5%), and in a pediatric ICU (73.2%). For nurses, the majority were females (76.0%), less than 30 years (< 82.3%), working 1– < 3 years in the specialty (30.1%), 1– < 3 years in the unit (45.3%) and working in a pediatric ICU (52.2%). The differences between physicians and nurses in age, sex, number of years in the specialty and type of ICU were statistically significant (*P* = 0.000, 0.003, 0.001, 0.001, 0.003, respectively) (Table 1).

**Table 1:** Sociodemographic characteristics of the ICU physicians and nurses, Egypt, 2024

Characteristics	Physicians *n* = 56		Nurses *n* = 408		Total *N* = 464		*P*-value
	No.	%	No.	%	No.	%	
Age							
< 30 years	18	32.1	336	82.3	354	76.3	0.000*
30–	36	64.2	62	15.1	98	21.1	
40–	1	1.8	8	2.0	9	1.9	
> 50	1	1.8	2	0.5	3	0.6	
Mean ± SD	31.58 ± 4.85		26.40 ± 4.68		27.03 ± 4.99		
Sex							
Male	24	42.9	98	24.0	122	26.3	0.003*
Female	32	57.1	310	76.0	342	73.7	
No. of years in the speciality							
< 6 months	1	1.8	56	13.7	57	12.3	0.001*
6 months– < 1 year	2	3.6	45	11.0	47	10.1	
1–	9	16.1	123	30.1	132	28.4	
3–	21	37.5	100	24.5	121	26.1	
5–	19	33.9	62	15.2	81	17.5	
10–	3	5.4	14	3.4	17	3.7	
> 20 years	1	1.8	8	2.0	9	1.9	
No. of work years in the unit							
< 6 months	1	1.7	14	3.4	15	3.2	0.001**
6 months– < 1 year	6	10.7	22	5.4	28	6.0	
1–	9	16.0	185	45.3	194	41.8	
3–	35	62.5	146	35.7	181	39.0	
5–	3	5.3	30	7.3	33	7.1	
10–	1	1.7	7	1.7	8	1.7	
> 21	1	1.7	4	1.0	5	1.1	
Type of ICU							
Adult	15	26.8	195	47.8	210	45.3	0.003*
Pediatrics	41	73.2	213	52.2	254	54.7	

^*^P value for chi-square test < 0.05

^**^P value for Fisher exact test < 0.05

The overall mean score of physicians and nurses’ attitude to patient safety was 63.7 ± 13.4 and percentage of positive responses was 58.1%. Job satisfaction was the dimension with the highest total mean and percentage of positive responses (70.0 and 65.1%, respectively). On the other hand, stress recognition was the dimension with the lowest mean score and percentage of positive responses (59.4 and 53.6%, respectively). Physicians attained a higher total mean score for the teamwork climate, job satisfaction, perceptions of management and work conditions domains (73.8 ± 10.4, 81.7 ± 14.6, 72.5 ± 19.4, 70.6 ± 18.1 respectively) than those of nurses (61.0 ± 15.4, 68.4 ± 23.0, 60.8 ± 22.4, 61.8 ± 22.3 respectively) and the difference was significant (*P* = 0.03, 0.00, 0.01, 0.00 respectively). Nurses had a significantly higher mean score of stress recognition (60.4 ± 23.6) than that of physicians (52.0 ± 24.0) (*P* = 0.01). Safety climate score was insignificantly higher among physicians (67.0 ± 12.7) than nurses (64.9 ± 16.7). The total mean score of physicians’ attitude (69.6 ± 9.9) was higher than that of nurses (62.9 ± 13.6) and the difference was significant (*P* = 0.04) (Table 2).

**Table 2:** Attitude of the ICU physicians and nurses to patient safety culture, Egypt, 2024

Safety attitudes domains	Physicians		Nurses		Total mean ± SD	Total positive responses (> 75%)	*P*-value
	Mean score ± SD)	Positive response (> 75%)	Mean score ± SD	Positive response (> 75%)			
Teamwork climate	73.8 ± 10.4	67.6	61.0 ± 15.4	27.8	62.5 ± 15.4	63.6	0.03 *
Safety climate	67.0 ± 12.7	62.0	64.9 ± 16.7	58.3	65.1 ± 16.2	54.7	0.24
Job satisfaction	81.7 ± 14.6	84.2	68.4 ± 23.0	62.5	70.0 ± 22.5	65.1	0.00*
Stress recognition	52.0 ± 24.0	59.3	60.4 ± 23.6	32.5	59.4 ± 23.8	53.6	0.01*
Perceptions of management	72.5 ± 19.4	69.3	60.8 ± 22.4	52.3	62.2 ± 22.4	54.3	0.01*
Work conditions	70.6 ± 18.1	72.3	61.8 ± 22.3	54.4	62.9 ± 22.0	56.6	0.00*
Total	69.6 ± 9.9	68.7	62.9 ± 13.6	57.7	63.7 ± 13.4	58.1	0.04*

^*^Independent sample *t*-tests *p* < 0.05

Regarding SAQ’s items mean scores, and percentage of positive answers; the dimension of job satisfaction yielded the highest mean score among both physicians and nurses (81.7 vs. 68.4 respectively). The highest rated statement was “I have the support I need from other personnel to care for patients,’’ with a mean score of 79.6 and the percentage of positive responses was 78.0%. On the other hand, the lowest-rated statement was ‘It is easy for personnel in this ICU to ask questions when they do not understand something,’ with a mean score of 33.0 and a positive response rate of 22.8%. For each dimension, the results of the internal consistency test revealed a rather high Cronbach α ranging from 0.712 for the dimension “Teamwork climate’’ to 0.855 for the dimension “job satisfaction’’ (Table 3).

**Table 3:** Mean scores, standard deviation and percentages of positive responses of ICU physicians and nurses for the items and scales in the Safety Attitude Questionnaire, Egypt, 2024

Item	Mean score	SD	% Positive responses > 75%
Teamwork climate (Cronbach α = 0.712)			
“Personnel input about patient care is well received in this ICU.’’	73.1	29.3	70.5
“It is easy for personnel in this ICU to ask questions when there is something that they do not understand’’	33.0	32.8	22.8
“The physicians and nurses here work together as a well-coordinated team’’	72.9	26.5	67.5
“Disagreements in this ICU are appropriately resolved’’	69.6	27.4	68.1
“I have the support I need from other personnel to care for patients.’’	79.6	26.4	78.0
“In this ICU, it is difficult to speak up if I perceive a problem with patient care’’	77.6	24.4	75.2
Safety climate (Cronbach α = 0.738)			
“I would feel safe being treated here as a patient’’	71.5	29.2	68.1
“I know the proper channels to direct questions regarding patient safety in this ICU’’	71.7	27.5	68.1
“I am encouraged by my colleagues to report any patient safety concerns I may have’’	65.8	28.0	61.0
“Medical errors are handled appropriately at this ICU.’’	65.3	26.8	55.6
“The culture in this ICU makes it easy to learn from the errors of others.’’	41.9	32.6	30.4
“I receive appropriate feedback about my performance’’	69.9	27.2	64.2
“In this ICU, it is difficult to discuss errors. “	69.8	27.4	35.8
Job satisfaction (Cronbach α = 0.855)			
“I like my job.’’	74.9	29.7	71.1
“I am proud to work at this hospital’’	71.7	27.2	68.5
“This hospital is a good place to work.’’	70.2	28.4	65.1
“Working in this hospital is like being part of a large family’’	70.6	27.9	67.2
“Morale in this ICU is high.’’	62.9	28.4	53.9
Stress recognition (Cronbach α = 0.708)			
“I am less effective at work when fatigued.’’	69.3	30.5	64.9
“When my workload becomes excessive, my performance is impaired’’	67.2	31.0	65.5
“I am more likely to make errors in hostile or tense situations’’	53.2	31.9	41.1
“Fatigue impairs my performance during emergency situations.’’	47.8	36.6	42.9
Perception of management (Cronbach α = 0.839)			
“Hospital management does not knowingly compromise the safety of patients.’’	51.9	34.2	42.0
“I am provided with adequate, timely information about events in this department that might affect my work.’’	66.2	30.8	57.5
“Hospital administration supports my daily efforts’’	65.4	31.3	60.1
“The levels of staffing in this ICU are sufficient to handle the number of patients.’’	64.4	29.1	56.3
“I get adequate, timely info about events that might affect my work, from hospital management’’	63.3	28.7	56.0
Working conditions (Cronbach α = 0.760)			
“This hospital does a good job of training new personnel.’’	59.3	32.4	54.3
“All the necessary information for diagnostic and therapeutic decisions is routinely available to me’’	68.0	28.4	63.6
“Trainees in my discipline are adequately supervised.’’	59.3	27.4	49.4
“This hospital deals constructively with problem personnel.’’	64.9	27.0	59.3

Job title was the only characteristic that showed significant difference between the participants where physicians had a statistically significant higher mean total safety attitude score (69.6 ± 9.9) compared to that of nurses (62.9 ± 13.6) (*P* = 0.000) (Table 4).

**Table 4:** Patient safety attitude’s overall mean score of the ICU physicians and nurses according to sociodemographic characteristics, Egypt, 2024

Characteristics	Mean ± SD	*P-*value
Age		
< 30	63.2 ± 13.5	0.502
30–	65.9 ± 13.3	
40–	61.2 ± 11.5	
> 50	57.5 ± 6.8	
Sex		
Male	61.8 ± 13.8	0.06
Female	64.4 ± 13.2	
No. of years in the speciality		
< 6 months	66.4 ± 12.6	0.05
6 months– < 1 year	63.7 ± 14.9	
1–	61.6 ± 14.9	
3–	63.3 ± 10.8	
5–	65.0 ± 14.2	
10–	65.8 ± 13.2	
≥ 20	68.2 ± 7.9	
No. of work years in the unit		
< 6 months	71.7 ± 12.5	0.45
6 months– < 1 year	64.0 ± 14.3	
1–	63.7 ± 13.3	
3–	63.3 ± 12.9	
5–	62.0 ± 16.7	
10–	64.4 ± 12.8	
≥ 20	63.5 ± 4.0	
Type of ICU		
Adult	64.2 ± 11.1	0.43
Paediatric	63.2 ± 15.0	
Job title		
Physicians	69.6 ± 9.9	0.00*
Nurses	62.9 ± 13.6	

^*^P value for independent sample *t*-tests and ANOVA test < 0.05

Comparing the results of the present study with the benchmark data from a study on 8,646 ICU professionals in UK, USA and New Zealand [18], the mean scores for the domains “job satisfaction’’, “perception of management’’ and “working conditions’’ in the current study were higher than those reported in the benchmark data. On the other hand, the mean scores for the dimensions “teamwork climate’’, “safety climate’’, and “stress recognition’’ were higher in the benchmark data compared to the current study (Table 5).

**Table 5:** Safety Attitudes Questionnaire results of the studied ICU physicians and nurses compared to benchmarking data, Egypt, 2024

Mean Scale Score (SD)	Benchmarking score^a^	Results of the present study
Teamwork climate	70.7 ± 18.6	62.5 ± 15.4
Safety climate	67.7 ± 17.0	65.1 ± 16.2
Job satisfaction	63.4 ± 21.6	70.0 ± 22.5
Stress recognition	65.9 ± 20.2	59.4 ± 23.8
Perception of management	48.0 ± 20.3	62.2 ± 22.4
Working conditions	58.6 ± 20.4	62.9 ± 22.0

^a^Benchmarking scores of the Safety Attitudes Questionnaire intensive care unit version (*n* = 8646) as published by Sexton et al. [18]

## Discussion

The current study aimed to assess patient safety attitude in the intensive care units of four public hospitals in Egypt. The mean scores for the three dimensions; “job satisfaction”, “perceptions of management”, and “work conditions” were higher compared to the benchmark data from a study conducted on 8646 ICU doctors and nurses in the UK, USA, and New Zealand. On the other hand “stress recognition’’, “teamwork climate’’, and “safety climate’’ were lower than the benchmark values. For the dimension “teamwork climate” the mean score for the current study was 62.5 ± 15.4 compared to 70.7 ± 18.6 in the benchmark study [18]. In another study conducted among healthcare workers in two ICUs in a University hospital in Egypt, teamwork within units attained the highest score among other safety culture dimensions [19]. Teamwork in critical care units has been linked to positive outcomes. For example, missed care was negatively and significantly correlated with teamwork in the ICU of three governmental hospitals in Egypt [20]. In 17 critical care units across the USA, staff members of the units with lower-than-expected mortality rates perceived their teams as being structured, organized, less dependent, more trustworthy, and functioning at a higher stage of group development than the staff members of the units with higher-than-expected mortality rates [21]. Hospitals can enhance teamwork by introducing efficient team interventions in the ICU, like rounds, standardising the rounding procedure with daily targets, and enhancing teamwork skills through team training [22].

The dimension “perception of management’’ scored higher than benchmark data (62.2 ± 22.4 compared to 48.0 ± 20.3, respectively) [18]. Similarly, the scores were higher compared to a study in Turkey (50.3 ± 30.6) and another study in five hospitals in Pakistan (51.3 ± 22.1 for physicians and 57.4 ± 21.1 for nurses) [23, 24]. The perception of management score was comparable to the findings of studies conducted among ICU staff in 16 NICUs in Palestine (63.2 ± 18.8), and in three general hospitals in Malaysia (64.87 ± 16.24) [25, 26]. Despite being higher than benchmark data and similar or higher than other studies, the perception of management domain in the current study was still moderately rated, compared to the other dimensions. Leadership commitment and support is essential for creating a patient safety climate in hospitals [27, 28]. One strategy to improve employee perceptions of management and in turn the safety culture, is senior executive walk rounds. The purpose of these walk rounds is to demonstrate senior leadership's dedication to patient safety [29, 30].

The dimension with the lowest mean score was “stress recognition’’ (59.4 ± 23.8). The scores were lower than those reported in the benchmark data (65.9 ± 20.2) as well as the scores from a study conducted in Egypt among primary care staff (63.7 ± 19.1) [1, 31], and it was much lower than the scores reported in surgical and medical wards in 21 Polish hospitals (71.6 for nurses and 80.86 for doctors) [32]. The domain of stress recognition differs from the other domains. The goal of this domain is to help individuals evaluate their performance in high-stress situations where they may be more likely to make mistakes. In contrast, the other domains elicit viewpoints on the workplace and the greater organizational unit [1]. One explanation of the low scores for stress recognition is that the highest percentage of nurses had 1– < 3 years of experience in the specialty and the highest percentage of doctors had 3– < 5 years in the specialty (30.1% and 37.5%, respectively). The increase in seniority was significantly associated with a decrease in stress recognition among doctors and nurses in Poland [32] and among nurses in Saudi Arabia [33]. Research has indicated that in difficult situations, nurses with less seniority tended to take a more task-oriented approach and were more likely to ask for help than those with more seniority. In order to manage stressful situations, less experienced nurses needed to exert more cognitive and behavioral effort [34]. Another explanation is that the professional culture in healthcare contributes to a lack of awareness of the impact of stress and exhaustion on performance as well as fostering a false sense of invulnerability.

A further argument is that compared to specialists in other departments, those working in the intensive care unit are more capable of handling the higher volume, acuity, and frequent incidence of hazards and inconveniences in their work. In a study conducted among Polish nurses in 6 ICUs, stress recognition domain was the domain with the least score (30.6 ± 14.4) with only one third of nurses recognizing the effect of fatigue on their performance in an emergency and only 10.7% agreeing that they were less effective when fatigued [35]. The domain “stress recognition’’ was the only domain in the current study where nurses scored significantly higher than physicians (52.0 ± 24.0 for physicians compared to 60.4 ± 23.6 for nurses) (Table 2). High levels of stress, heavy workloads, and a lack of sleep have been shown to impair judgement and raise the risk of medical errors [36]. It is recommended to put into practice a number of measures, such as setting work and rest schedules, developing stress management policies, and promoting healthy living through health programs [37].

Physicians had significantly higher safety scores in the overall score as well as in all individual domains, except for stress recognition (Tables 2 and 3). The findings of previous studies on this aspect were not consistent. In a pediatric surgical intensive care unit at a Dutch university hospital, nurses consistently had higher mean scale scores compared to doctors [38]. Similarly, in 16 NICUs in the West Bank, Palestine, nurses scored slightly higher on safety culture than physicians although it was not statistically significant [25]. On the other hand, in a study conducted among NICU caregivers in a sample of 12 hospitals in the USA, physicians’ safety climate scores were significantly higher than nurses and ancillary personnel [39]. Another study compared the views of critical care physicians and nurses in eight ICUs in the USA on teamwork. The nurses reported that it is hard for them to speak up, that disagreements are not effectively addressed, that they need greater input into decision-making, and that their advice is not well received [40]. Similarly, nurses attained lower overall safety climate scores compared to physicians in a study conducted in 92 hospitals in the USA. The results of this study suggest that nurses may be more acutely aware of safety flaws than physicians because of their closer working relationship with the institution [41]. Thus, it is important to stratify the results of longitudinal surveys by job category to avoid overestimation of scores if, for example, only physicians were surveyed [39].

The domain “job satisfaction’’ was the domain with the highest scores in the current study (total mean score of 70.0 ± 22.5 and percentage of positive response of 65.1%). This finding aligns with the results of studies in ICU units in Palestine, Cyprus and the USA where the satisfaction domain attained the highest scores across all domains [9, 19, 26]. The high score of the satisfaction domain in the current study can be attributed to the small size of surveyed ICUs ranging from 16 to 50 beds. Working in small ICUs where everybody knows each other can help mitigate working conditions effects. In addition, because ICUs frequently give employees a sense of status, working there can lead to greater job satisfaction [35]. The satisfaction domain showed the widest difference in score between physicians and nurses (81.7 ± 14.6 for physicians compared to 68.4 ± 23.0 for nurses). Job satisfaction can be considered an outcome of other safety climate domains, specifically the teamwork domain where nurses scored significantly lower than physicians. Teamwork was associated with greater job satisfaction in previous research [42]. Since job satisfaction has been linked to higher productivity and lower turnover among nurses, hospital managers should implement teamwork training programs, especially among nurses, and measure the effectiveness of this training.

The individual items with the lowest mean scores were “It is easy for personnel in this ICU to ask questions’’ followed by “The culture in this ICU makes it easy to learn from the errors of others’’ (41.9 ± 32.6). This result is consistent with several other studies conducted in Egypt. In a study conducted in a university hospital in Cairo, the lowest score for safety culture was attributed to the dimension of non-punitive response to error. This result was similar to results reported in a gynecology and Obstetrics University hospital in Alexandria and in two ICUs in a University hospital in Alexandria [14, 15]. Moreover, in a systematic review investigating safety culture in Arab countries, all participants in all countries rated non-punitive response to error negatively suggesting that a culture of medical dominance is inherent in the organizational culture of the health systems in Arab countries [43]. Hospital management should make sure that accountability is accompanied with an avoidance of blame and negativity to combat the culture of blame and medical domination. Furthermore, in order to assist healthcare organizations in identifying patient risks and learning from their mistakes, laws and regulations should be put in place to guarantee the creation of patient safety reporting systems [43].

### Strengths and limitations

Using the SAQ, a validated instrument for evaluating safety climate, is one of the study's strengths. The tool is a reliable psychometric tool for evaluating six climatic domains relevant to safety. Since it has been demonstrated that the six domains show less variance when utilized at the clinical area level as opposed to the hospital, the instrument is suited to evaluate safety culture for professionals working in intensive care units. The availability of public benchmarking data for the SAQ, which allowed us to compare our own climatic data with the benchmark data, is another area of strength. The SAQ's widespread application also makes it possible to track changes over time in longitudinal surveys as well as conduct reliable comparisons across hospitals, patient care regions, and types of caregivers. Another strength of the study is that the reliability of the current administration of the tool was assessed using Cronbach’s α, which ranged from 0.712 to 0.855 indicating good reliability.

A limitation of the current study is that the study was only conducted in public hospitals excluding the professionals in the private sector. The public hospitals in the current study also did not represent all types of public hospitals as health insurance hospitals were not included. Thus, the results cannot be generalized outside ICU settings at Ministry of Health and University Hospitals. Another limitation is the use of a self-reported questionnaire which could have resulted in a social desirability bias and an overrating of the domains.

## Conclusion

The current study showed that in the studied ICUs some safety culture domains were better when compared to benchmark data. Others have room for improvement such as “stress recognition’’, “teamwork climate’’ and “safety climate’’ which highlights the importance of targeting safety climate improvement interventions and tracking the improvement through continuous measurement.

## Data Availability

The data set for the current study is available upon reasonable request to the corresponding author.

## Abbreviations

SAQ: Safety Attitudes Questionnaire
ICU: Intensive care units
DALYs: Disability-adjusted life years
GAHAR: The General Authority for Healthcare Accreditation
NICU: Neonatal intensive care unit
MOHP: Ministry of Health and Population
I-CVI: Item Content Validity Index
ANOVA: Analysis of variance
